# Computational and experimental advances in liver-on-a-chip technology for cancer research: a systematic review

**DOI:** 10.1007/s12551-024-01260-z

**Published:** 2024-12-14

**Authors:** Violeta Carvalho, Mariana Ferreira, Raquel O. Rodrigues, Senhorinha F. C. F. Teixeira, Rui A. Lima

**Affiliations:** 1 MEtRICs, Mechanical Engineering Department, University of Minho, Campus de Azurém, 4800-058 Guimarães, Portugal; 2 ALGORITMI/LASI Center, University of Minho, Campus de Azurém, 4800-058 Guimarães, Portugal; 3 Center for MicroElectromechanical Systems (CMEMS-UMinho), University of Minho, Campus de Azurém, 4800-058 Guimarães, Portugal; 4 LABBELS—Associate Laboratory, Braga/Guimarães, Portugal; 5 Advanced (magnetic) Theranostic Nanostructures Lab, Nanomedicine Unit, INL–International Iberian Nanotechnology Laboratory, Av. Mestre José Veiga, 4715-330 Braga, Portugal; 6 CEFT - Transport Phenomena Research Center, Faculty of Engineering, University of Porto, Rua Dr. Roberto Frias, 4200-465 Porto, Portugal; 7 ALiCE - Associate Laboratory in Chemical Engineering, Faculty of Engineering, University of Porto, Rua Dr. Roberto Frias, 4200-465 Porto, Portugal

**Keywords:** Liver-on-a-chip, Numerical simulation, Mathematical model, Hepatocellular carcinoma

## Abstract

The integration of numerical simulations with Liver-on-a-Chip (LoC) technology offers an innovative approach for studying liver physiology and pathology, especially in the context of liver cancer. Numerical simulations facilitate the optimization of microfluidic devices’ design and deepen the understanding of fluid flow and mass transfer. However, despite significant advancements, challenges such as replicating the full complexity of the liver microenvironment and scaling up for high-throughput screening persist. This systematic review explores the current advancements in LoC devices, with a particular emphasis on their combined use of numerical simulations and experimental studies in liver cancer research. A comprehensive search across multiple databases, including ScienceDirect, Wiley Online Library, Scopus, Springer Link, Web of Science, and PubMed, was conducted to gather relevant literature. Our findings indicate that the combination of both techniques in this field is still rare, resulting in a final selection of 13 original research papers. This review underscores the importance of continued interdisciplinary research to refine these technologies and enhance their application in personalized medicine and cancer therapy. By consolidating existing studies, this review aims to highlight key advancements, identify current challenges, and propose future directions for this rapidly evolving field.

## Introduction

Cancer is a complex disease characterized by several hallmarks, including sustained proliferative signaling, evasion of growth suppressors, resistance to cell death, replicative immortality, induction of angiogenesis, and activation of invasion and metastasis (Hanahan and Weinberg 2011; Hanahan 2022). These hallmarks, first defined by Hanahan and Weinberg, provide a framework for understanding the multifaceted nature of cancer progression and its resistance to therapy (Hanahan and Weinberg 2000). Liver cancer exemplifies these characteristics and poses a significant global health burden, characterized by a high mortality rate that is expected to increase by 6.7% by 2025 (WHO 2024a, 2024b). Hepatocellular carcinoma (HCC), which constitutes up to 80% of liver cancer cases, is frequently diagnosed at an advanced stage, limiting treatment options (Rumgay et al. 2022a, 2022b; McGlynn et al. 2021). Despite advancements in diagnostic and therapeutic strategies, the prognosis for liver cancer remains poor, with a five-year survival rate of less than 20% for advanced-stage diagnoses (Yang and Roberts 2010; Calderon-Martinez et al. 2023). This highlights the critical need for innovative approaches in disease modeling and drug development.

Current treatments for liver cancer include surgical resection (Imamura et al. 2003), liver transplantation (Mazzaferro et al. 1996), radiofrequency ablation (Vakili et al. 2009), transarterial chemoembolization (TACE) (Lo et al. 2002), radioembolization (Sangro et al. 2011), immunotherapy (Sangro et al. 2021), and systemic therapies (Kudo et al. 2018; Panagiotis and Chau 2024). While these treatments can be effective, they often come with significant limitations. Surgical options are only viable for a small subset of patients with early-stage disease, and liver transplantation is limited by donor availability and the risk of recurrence (Llovet et al. 2021). Furthermore, radiofrequency ablation and transarterial chemoembolization may not be suitable for advanced-stage tumors, and systemic therapies frequently result in severe side effects and may only extend survival by a few months (Forner et al. 2018; Rezaei 2023). These challenges emphasize the pressing need for more effective and less toxic treatment options for liver cancer.

In response to these limitations, researchers are exploring alternative treatments, including novel targeted therapies (Huang et al. 2020; Li et al. 2022a; Zhao et al. 2022; Seidi et al. 2022; Moukhtari et al. 2023), gene therapies (Hernández-alcoceba et al. 2007; Reghupaty and Sarkar 2019; Kohn et al. 2023), and combination treatments to improve outcomes for liver cancer patients (Liu et al. 2021; Taha et al. 2022; Gao et al. 2015). However, evaluating the efficacy and safety of these new approaches requires robust preclinical testing. This is where *in vitro* models become crucial (Hrout et al. 2022; Blidisel et al. 2021; Yao et al. 2021; Chen et al. 2015; Bonanini et al. 2022). Traditional *in vitro* and *in vivo* models, although valuable, exhibit limitations in accurately replicating the human microenvironment and predicting patient responses to therapy. Conventional 2D cell cultures, for instance, fail to reproduce the complex 3D architecture and physiological conditions of human organs, leading to poor translational outcomes (Godoy et al. 2013; Kapałczyńska et al. 2018). Similarly, animal models, while offering a systemic context that can be informative, often fail to accurately replicate the complexity of the disease, including tumor heterogeneity, immune interactions, and the dynamic nature of the organ’s microenvironment. Furthermore, their use raises significant ethical concerns (Kiani et al. 2022; Atat et al. 2022). To bridge the gap between conventional models and human physiology, advanced *in vitro* systems such as 3D spheroid cultures, organoids, and microfluidic-based Organ-on-a-Chip (OoC) technology have emerged as promising platforms (Maia et al. 2022; Carvalho et al. 2021a; Prodanov et al. 2016). Spheroids and organoids offer enhanced cellular interactions and more accurate functional replication of human tissue compared to 2D cultures (Nguyen et al. 2021; Luo et al. 2024; Lee et al. 2021; Liu et al. 2023). However, while these systems offer advantages over traditional models, they still face challenges in fully replicating the complexity of the cancer’s microenvironment and its interactions with other organ systems (Harane et al. 2023; Arora et al. 2024). These models often operate under static conditions, whereas the human body exists in a dynamic environment with constant fluid flow, mechanical forces, and evolving cellular interactions. As a result, they may fall short in accurately modeling tumor behavior, and drug responses. Furthermore, spheroids and organoids often lack the full range of cell types and the native extracellular matrix (ECM) found in organs, which limits their physiological relevance. In contrast, organotypic cultures offer a more comprehensive cell population and native ECM structure, closely mimicking *in vivo* tissue architecture and function (de Hoyos-Vega et al. 2023; Hattersley et al. 2011, 2008). OoC technology has further advanced the field by integrating microfluidics and 3D culture models to more closely mimic the *in vivo* environment (Liu et al. 2022a; Carvalho et al. 2022; Sontheimer-Phelps et al. 2019; Driver and Mishra 2023). These devices typically consist of a network of microchannels and chambers that can include multiple cell types, extracellular matrix components, and fluid flow to closely mimic cellular heterogeneity and structural complexity. Additionally, a key advantage of microfluidic devices is their ability to facilitate the accumulation and controlled circulation of endogenous growth factors, which allows cells to better retain their phenotype over extended periods compared to conventional static cultures. This accumulation of growth factors, alongside dynamic fluid flow, enhances cellular function and viability, offering a more stable and physiologically relevant environment. Recent studies have explored how hepatic growth factors accumulate and are secreted within these devices, underscoring the value of microfluidics in sustaining cell-specific functions and advancing tissue modeling (Lu et al. 2021; Zhou et al. 2015; Hoyos-Vega et al. 2024). Because of all these characteristics, OoC technology has rapidly evolved since its inception in 2010 (Huh et al. 2010). This pioneering work introduced a lung-on-a-chip device, which successfully replicated the critical functions of the human alveolar-capillary interface. Since then, the field has rapidly evolved, leading to the development of various organ-specific chips, including Liver-on-a-Chip (LoC) devices (Mirzababaei et al. 2023; Lee et al. 2019; Polidoro et al. 2024; Xie et al. 2022), which have been used for diverse purposes such as investigating the effects of substances on gene expression profiles (Solan et al. 2023), studying metabolomic interactions between liver sinusoidal endothelial cells and hepatocytes (Tian et al. 2022; Messelmani et al. 2024, 2023), evaluating the genotoxicity and mutagenicity of drugs (Kopp et al. 2024), impact of proteins in liver cancer progression (Shen et al. 2023), study cardiotoxicity (Soltantabar et al. 2021), cell resistance (Poddar et al. 2024), drug toxicity (Kim et al. 2022; Lee et al. 2023), cell separation/isolation (Xue et al. 2016; Chen et al. 2017a, 2020, 2017b; Rodríguez-Pena et al. 2022), and detection purposes (Deepak et al. 2024).

The integration of computational simulations with OoC systems presents a powerful approach to accelerate research by offering predictive tools that complement experimental studies and help improve device performance for specific applications (Carvalho et al. 2021b). This typically involves creating a virtual representation of the microchannel network, and model interactions between diverse elements: flow rates, materials properties, cellular properties (e.g., molecular consumption), design factors, and other conditions such as temperature, pressure, oxygen levels, pH, and diffusion rates for various solutes, including nutrients and growth factors, among others (Choe et al. 2017; Jeon et al. 2020). Generally, the modeling process involves initial design, mesh generation, defining flow conditions, material properties, and boundary conditions, simulation, and whenever possible, validation against experimental data. By enabling precise control over critical parameters, such as flow dynamics and shear stress, computational modeling can serve as a powerful predictive tool for assessing the impact of design variations and experimental conditions (e.g., nutrient levels and availability). These predictive insights encompass the ability to anticipate system behavior, such as how specific design changes or parameter adjustments will affect device performance or replicate physiological conditions. In doing so, computational modeling minimizes reliance on extensive trial-and-error experimentation, accelerating the development process. While the initial investment in computational tools, such as hardware and software licenses, may be substantial, these tools deliver long-term cost savings by reducing the need for consumables, manual labor, and repeated physical prototyping. Although specific cost comparisons depend on the context, the scalability and reusability of computational tools typically result in cumulative cost efficiency over multiple tests (Carvalho et al. 2024; Solovchuk et al. 2013; Li et al. 2022b; Zhang et al. 2021). Moreover, computational modeling facilitates an *in silico* high-throughput approach to testing diverse design scenarios and parameter configurations. This allows researchers to rapidly evaluate multiple configurations *in silico*, systematically identifying promising designs or conditions for experimental assays. Finally, by integrating computational modeling with experimental data, researchers can iteratively refine and validate their models. This integration enhances predictive accuracy and reliability, ensuring that the insights generated align closely with experimental outcomes.

Fluid flow dynamics simulations are widely used in OoC systems’ research to enhance microfluidic channel geometries, flow rates, and shear stress parameters, enabling researchers to mimic the mechanical cues that cells experience *in vivo* more accurately (Saeedabadi et al. 2022; Jun-Shan et al. 2017). Similarly, modeling nutrient and oxygen gradients is crucial for recreating tissue-like microenvironments in OoC devices, as it helps ensure effective nutrient distribution across cell layers and improve the device to provide adequate levels for the experiments, creating a more physiologically relevant platform for cell culture (Wang et al. 2018). Additionally, computational models are helpful in simulating the transport, and efficacy of therapeutic agents within OoC systems. These predictive models enable researchers to assess drug distribution and diffusion kinetics within tissue analogs, refining both device parameters and experimental protocols (Barisam et al. 2017; Yang et al. 2022).

Common computational approaches in the OoC field include the Finite volume method (FVM), and finite element modeling (FEM). FVM divides the simulation domain into control volumes and applies conservation laws for mass, momentum, and energy directly over each volume (Versteeg and Malalasekera 2007), and is commonly implemented in software such as ANSYS Fluent. On the other hand, FEM breaks down the domain into elements and uses interpolation functions to approximate the solution, which is advantageous for complex geometries and varying material properties (Liu et al. 2018). FEM is often applied in OoC to model structural mechanics, such as tissue deformation due to its flexibility with complex boundaries. COMSOL Multiphysics is a popular FEM-based software in these applications.

This holistic perspective accelerates translational research by enabling the development of more effective preclinical models. However, significant challenges hinder their full potential, as outlined in Table 1.

**Table 1:** Advantages and challenges of integrating computational simulations with LoC technology

Advantages	Disadvantages
Predictive insights	Complexity in modeling
Customization for specific applications	Computational resources
Enhanced device design	Validation issues
Reduction in experimental costs	Over-simplification
High-throughput approach	Generalization limitations
Integration with experimental data	Lack of standardization

Accurately modeling complex biological environments, such as the liver, is challenging due to its complex microarchitecture, diverse cell types, and spatially varying oxygen and nutrient levels. Simulating these elements alongside dynamic cellular signaling and metabolic reactions requires advanced models and substantial computational resources, potentially limiting their accessibility to many research labs.

Furthermore, validating computational models for OoC research becomes more costly and challenging when incorporating cellular components, as complex experiments are often difficult to fully replicate *in silico*, and model simplifications are required. For instance, simplifying material properties (both device and ECM), cellular behaviors, molecular diffusion coefficients, and assuming ideal conditions (e.g., Newtonian fluids, laminar flow, smooth channels) can compromise the physiological relevance of model predictions.

Moreover, generalizing computational results across studies remains challenging due to variations in methods, parameters, and biological contexts. Thus, findings from a specific model may not apply broadly due to restricted study conditions. Lack of standardization, however, refers to inconsistencies in methods, protocols, and parameters across studies, leading to discrepancies that make comparisons and interpretations difficult.

Addressing these challenges is crucial to maximizing the impact of computational models in advancing translational research.

Although the potential of integrating LoC technology with computational modeling is evident, a comprehensive evaluation of this combined approach in liver cancer research is still lacking. Furthermore, despite several reviews addressing LoC devices for various applications (Ortega-Ribera 2024; Xie et al. 2023; Aina 2023; Deng et al. 2019), they often overlook the integration of computational tools. This systematic review aims to bridge this gap by thoroughly assessing the current state of LoC devices designed to replicate HCC, the most common type of primary liver cancer, and evaluating their use in conjunction with computational modeling. To the best of the authors’ knowledge, no previous systematic review has explored this integration in detail.

## Methodology

### Literature search

A comprehensive literature search was conducted across ScienceDirect, Wiley Online Library, Scopus, Springer Link, and PubMed using the following query ((("liver-on-a-chip" OR "liver chip" OR "microfluidic device") AND ("numerical simulation" OR "mathematical model" OR simulation) AND ("liver cancer" OR "hepatocellular carcinoma" OR "hepatocelular carcinoma"))). The search was conducted on 5^th^ July 2024.

### Eligibility criteria

The inclusion criteria for this review were original research articles that combine numerical and experimental approaches in LoC devices for cancer research and full-text articles. The exclusion criteria included abstracts, non-English language articles, literature reviews, systematic reviews, meta-analyses, book chapters, short communications, conference articles, patents, case reports, and books.

### Data analysis

After selecting the articles from the databases, they were organized in an Excel sheet to remove duplicates. Two authors independently conducted the same selection process, and then compared and discussed their results until reaching a consensus. If uncertainties remained about the inclusion or exclusion of certain studies, a third author was consulted to provide input and assist in the final decision. Based on their reviews and analysis of the inclusion and exclusion criteria, a final selection of relevant studies was made.

## Results and discussion

This section presents the PRISMA flowchart, outlining the paper selection process, followed by a detailed analysis and discussion of the selected studies and a table summarizing their key findings.

### Study selection

**Fig. 1:**
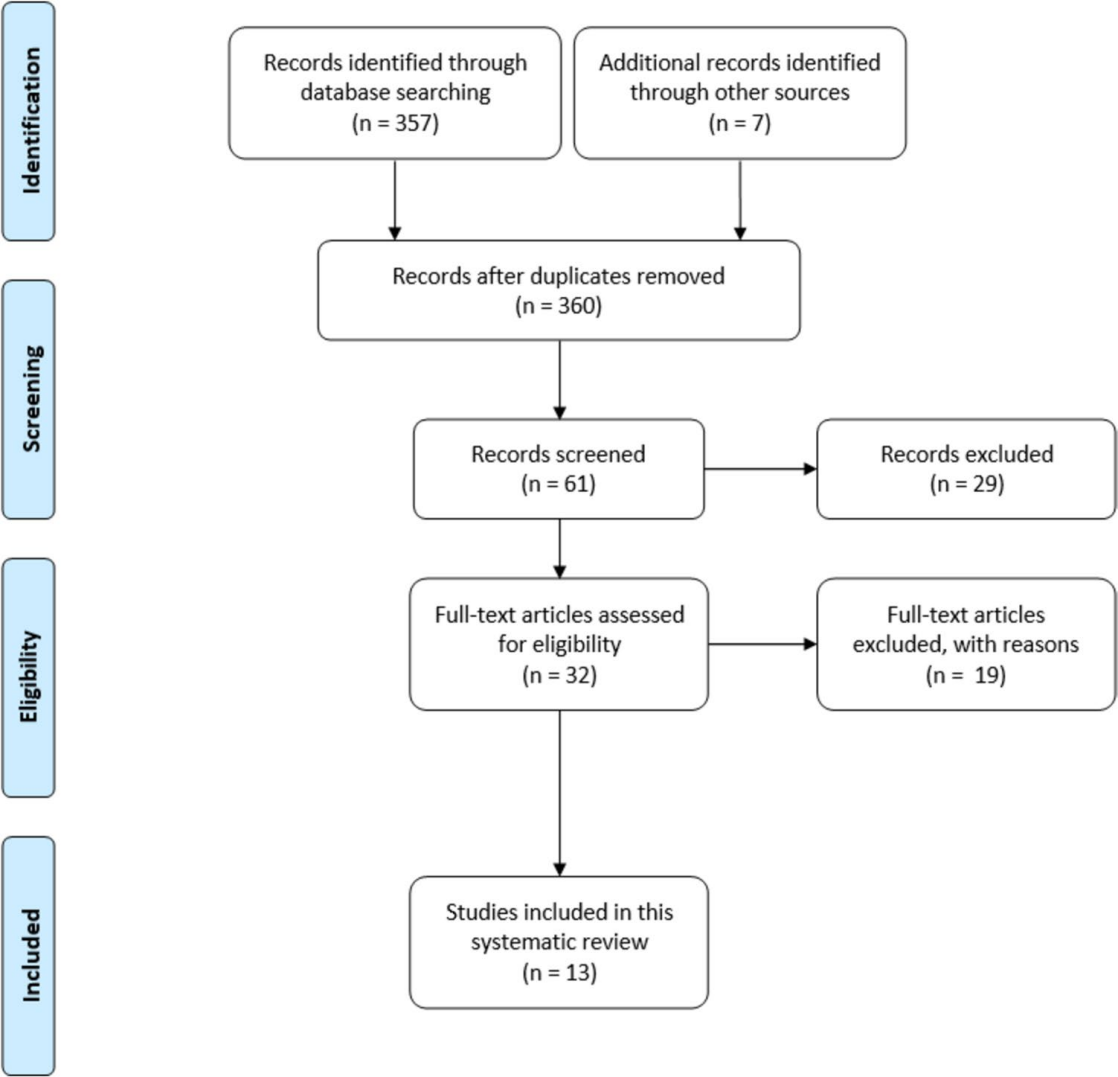
PRISMA flow diagram illustrating the study selection process

**Fig. 2:**
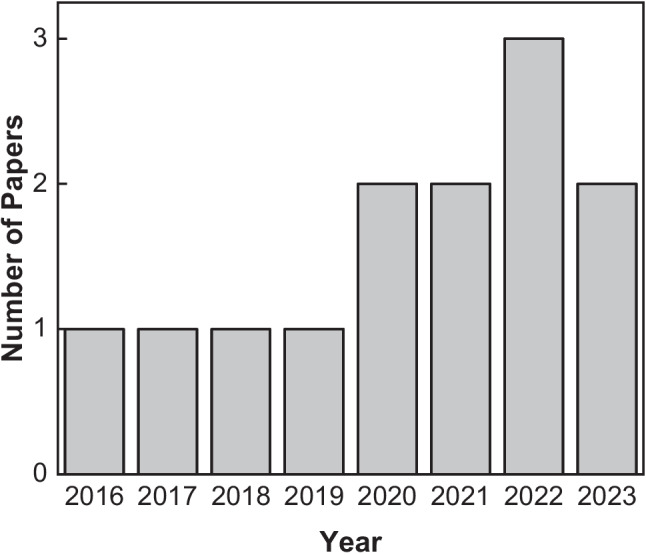
Number of papers included in the systematic review by year of publication

In the ScienceDirect database, 58 research articles were identified. PubMed, Scopus, and Web of Science each provided 2 results. The Wiley Online Library had 270 results, though many were review papers due to the inability to filter by paper type on the website. The search on SpringerLink returned 23 results, and 7 additional papers were identified from other sources. After removing duplicates, a total of 360 articles were reviewed. The initial selection was based on title screening, resulting in 61 articles that met the criteria for this systematic review. Next, abstracts of these 61 articles were evaluated, and 32 were selected for further review through full-text reading. Following a comprehensive screening of the full texts, 13 articles were selected for inclusion in the study. Figure 1 illustrates the selection process, and Fig. 2 displays the distribution of the published papers by year.

### Integrating LoC technology with computational simulations

The integration of computational simulations and experimental approaches has significantly advanced the development of LoC models for cancer research. These sophisticated platforms can better replicate the complex liver microenvironment, facilitating precise drug testing and enhancing our understanding of liver physiology and disease mechanisms.

**Fig. 3:**
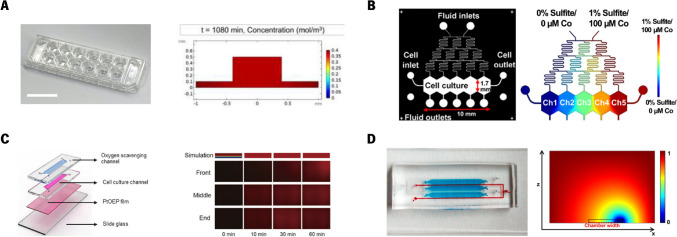
Representation of the devices developed by the authors and their numerical outputs. (**a**) A 3D *in vitro* tumor vasculature model for HCC in a hypoxia incubator, and oxygen concentration after 1080 min. Reproduced with permission (Lim et al. 2021). Copyright 2021, Wiley. Open Access; (**b**) A microfluidic platform to generate continuous oxygen gradients and sulfite concentration distribution. Reproduced with permission (Kang et al. 2020, 2018). Copyright 2020, Wiley. Open Access, and Copyright 2018, Nature, Open Access; (**c**) Microfluidic device developed to create hypoxic conditions, and the oxygen concentration distribution over time obtained numerically. Reproduced with permission (Kim et al. 2023). Copyright 2023, Elsevier. OpenAccess, (**d**) Microfluidic device to establish an oxygen gradient, which directs the metabolic zonation, and the steady-state oxygen concentration obtained from the numerical simulations. Reproduced with permission (Tonon et al. 2019). Copyright 2019, Nature. OpenAccess

Some authors (Lim et al. 2021; Kang et al. 2020) have focused on replicating the tumor microenvironment, particularly under hypoxic conditions. Hypoxia is a common feature of the tumor microenvironment (TME) and plays a significant role in cancer progression. Under these conditions, cancer cells adapt and can contribute to tumor progression processes including tumor angiogenesis and proliferation, and become chemo-resistant. By replicating hypoxia in research models, researchers can better understand how tumors adapt and survive in low-oxygen environments, which is essential for developing more effective therapies. In light of this, Lim et al. (2021) developed a 3D *in vitro* tumor vasculature model for HCC in a hypoxia incubator (Fig. 3(a)).

The results demonstrate that hypoxic stress in HCC vasculature promotes angiogenesis, HIF-1 expression, cell proliferation, and drug resistance, closely mimicking physiological tumor responses, making the microfluidic platform a promising tool for developing anticancer therapies targeting hypoxic tumor environments. Furthermore, the authors modeled the oxygen distribution within the platform to assess how external conditions influence the TME of HCC. The findings demonstrated that within 18 h, the external conditions fully compensated for the oxygen levels throughout the TME, providing a detailed spatial distribution of oxygen across the platform. In the same line of research, Kang et al. (2020) advanced the field by designing a microfluidic platform to generate continuous oxygen gradients, simulating both physiological and severe hypoxic conditions (0.3–6.9%), using a chemical oxygen‐quenching approach (Fig. 3(b)). The results confirmed that an ascending hypoxia gradient in hepatocytes led to decreased cell viability, increased reactive oxygen species accumulation, and higher HIF-1α expression, indicating distinct metabolic and genetic responses to varying oxygen levels. Herein, authors simulated sodium sulfite concentration gradients in the device, establishing well-separated compartments with varying concentrations by adjusting flow rates from 30 to 120 µL/h, and considering a diffusion coefficient of 1.2 × 10^−9^ m^2^/s in water, and a molecular weight of 126 Da. The authors experimentally validated their computational model in a previous study (Kang et al. 2018). In this case, they also tested different flow rates, but later fixed one flow rate, and evaluated the effect of different diffusion coefficients representing different species (3-MC, glucagon, and insulin) in the concentration profile. For validation purposes, they conducted laboratory experiments to test the impact of volumetric flow rate and molecular weight of inducers on the gradient profile. Initially, they visualized gradient formation in their microfluidic device using food colorings. Subsequently, they replicated their computer simulations, using three fluorescently labeled particles with varying molecular weights, and evaluated how the gradient profile matched the simulation predictions. The experimental results showed good agreement with the computational studies, although some imaging artifacts were noted. On the other hand, since albumin is a key biomarker of liver function, Kim et al. (2023) developed a hepatic hypoxia-on-a-chip with an electrochemical albumin sensor to investigate liver function changes under hypoxic conditions (Fig. 3(c)). This contains an oxygen-scavenging channel with a gas-permeable membrane in between, to quickly induce hypoxia (below 5% within 10 min). Results showed a significant decrease in albumin secretion (27% reduction) under hypoxic conditions after 24 h compared to normoxia, aligning with *in vivo* studies. This system provides a promising approach for real-time monitoring of liver function in response to hypoxia. Computational simulations were conducted to evaluate the system's ability to induce hypoxia over time. A no-slip boundary condition was applied at the channel boundary (except for the polydimethylsiloxane (PDMS) membrane). The cell culture medium was modeled as air-saturated water, using an incompressible laminar flow at 100 µL/h, with a density of 1 g/cm^3^ and viscosity of 1 cP. In the oxygen-scavenging channel, water with 0% oxygen concentration flowed at 100 µL/min. Furthermore, a free and porous media flow model was applied for oxygen transport across the PDMS membrane, with a porosity of 1.0 × 10^−6^ and a permeability of 620 Barrer. The authors specifically assessed how the position of the oxygen scavenging channels influenced oxygen concentration in different regions of the cell culture channel, using numerical simulations to compare the two design variations.

From a different perspective, Tonon and co-workers (2019) introduced a novel approach by designing a microfluidic device composed of culture chambers and gas channels. This allows to establish an oxygen gradient, which directs the metabolic zonation (spatial variation in metabolic functions and gene expression across different regions of the liver) of human embryonic stem cells (hESCs) into differentiated hepatocytes, thereby replicating *in vivo* conditions (Fig. 3(d)). The results showed that the microfluidic device successfully generated an oxygen gradient that guided the differentiation of hESCs into hepatocytes, with high oxygen levels enhancing glycogen storage and specific gene expression related to liver function. Conversely, low oxygen conditions increased the expression of genes associated with metabolic activities and cell proliferation, indicating that the device can effectively simulate metabolic zonation and improve the understanding of hepatocyte physiology in varying oxygen environments. The authors numerically simulated the oxygen concentration over time within the microfluidic chip under conditions similar to standard normoxic biological incubators and accounting for cell oxygen consumption. The computational model was designed to simulate the steady-state effect of gas concentration in channels, using diffusion with flux, insulation, and concentration boundary conditions. The portion simulated consists of a culture chamber and gas chambers, designed to operate in a normoxic environment. Due to PDMS’s high gas permeability, stable oxygen levels were generated by diffusion across a membrane that separates gas and flow chambers. Additionally, the impact of insulating the top of the chip on concentration gradients was examined. At higher gas flow rates (> 0.1 mL/min), the oxygen concentration was stable across the channel length, equaling the inlet concentration. Oxygen diffusion coefficients used were: PDMS (4 × 10^–9^ m^2^/s), phosphate-buffered saline (2 × 10^–9^ m^2^/s), and air (2 × 10^–5^ m^2^/s). Experimentally, they used a ruthenium solution as a fluorescence sensor for oxygen concentration. The experimental oxygen profile matched the predictions from the computational model, confirming the simulation's accuracy and demonstrating that the chip could effectively create and maintain different oxygen zones within the culture chamber. By accurately mimicking the physiological conditions within the liver, such models can provide deeper insights into how cells interact with their environment, adapt to different metabolic zones, and respond to therapies.

**Fig. 4:**
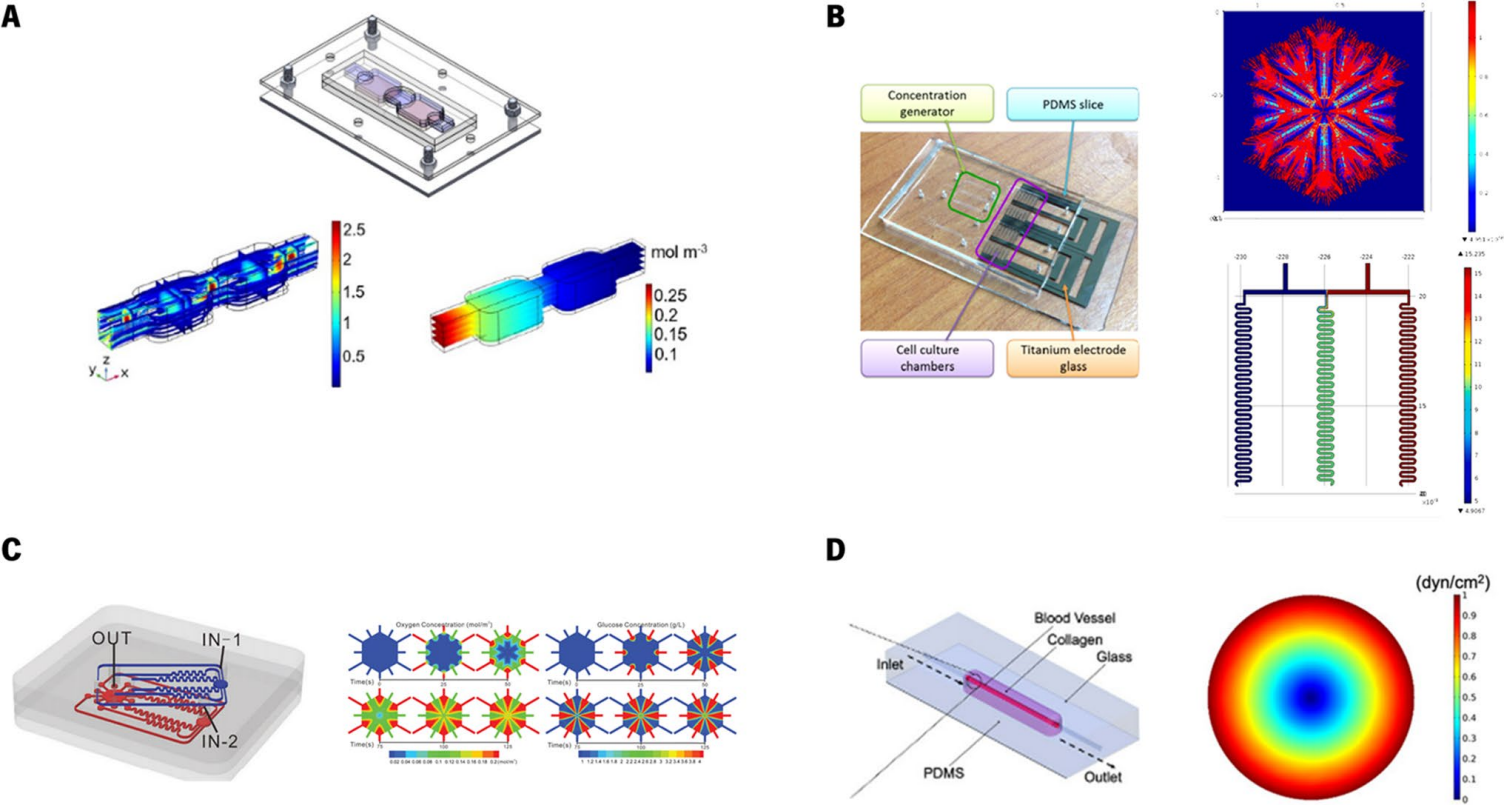
Devices and numerical outputs obtained by the authors. (**a**) HCC–bone metastasis assembled bioreactor, computed velocity streamlines within the device, and oxygen concentration distribution. Reproduced with permission (Sharifi et al. 2020). Copyright 2020, Springer, (**b**) Liver-lobule-mimicking labchip, and simulations of electric-field distribution, and GelMA concentration gradient. Reproduced with permission (Chen et al. 2021). Copyright 2021, Elsevier, (**c**) A biomimetic LoC with vascularized liver tissue, Tri-Vascular Liver-on-a-Chip. The numerical outputs show the oxygen and glucose concentration distribution. Reproduced with permission (Liu et al. 2022b). Copyright 2022, Frontiers. OpenAccess, (**d**) 3D vascularized HCC-on-a-chip model, and simulated shear stress. Reproduced with permission (Özkan et al. 2023). Copyright 2023, Wiley. OpenAccess

Other studies have integrated multi-organ interactions to explore drug metabolism and potential side effects. Kamei et al. (2017) developed an integrated Heart/Cancer-on-a-Chip to study the cardiotoxic effects of doxorubicin (Dox), an anti-cancer drug. The device enables the modeling of cardiotoxicity caused by Dox, where toxic metabolites produced by HepG2 cells (liver cancer cell line) can affect heart cells through the circulatory system. The device comprised a perfusion layer with two cell culture chambers and microchannels for nutrient and drug supply and a control layer with pneumatic valves and peristaltic micro-pumps for independent cell culture and precise medium flow control. Computational simulations were used to simulate the hydraulic performance of PDMS membrane to determine its threshold hydraulic pressure. The simulation results, showing a threshold pressure of 65 kPa, were compared with experimental measurements of valve efficiency and flow rate, demonstrating that the simulated and measured threshold pressures were in close agreement (less than 3% difference). Additionally, fluorescent imaging confirmed that the pneumatic valve was fully closed at this threshold pressure of 65 kPa. On the other hand, Sharifi and co-workers (2020) developed a metastasis-on-a-chip platform designed to model and track the spread of HCC to bone tissue (Fig. 4(a)). The platform consists of two main chambers, one containing encapsulated liver cancer cells and another simulating a bone environment. A microporous membrane mimicking the vascular barrier is placed above these chambers. The authors observed that liver cancer cells proliferated within the tumor microenvironment and eventually migrated through the circulatory system into the bone chamber. To investigate potential treatments, the researchers tested an herb-based compound, in both free form and encapsulated in nanoparticles and the results showed that the nanomaterial provided a more sustained inhibitory effect on cancer cell migration compared to the free form. Overall, the HCC-bone metastasis-on-a-chip platform successfully modeled key aspects of cancer metastasis, demonstrating its potential for studying metastasis-related biology and screening anti-metastatic drugs. In addition to the experimental observations, the study incorporated numerical simulations to model fluid dynamics and oxygen transport within the bioreactor. The culture medium was modeled as a steady, incompressible, isothermal, Newtonian fluid. To determine the velocity distribution in the chamber and hydrogel matrix, the continuity, Navier–Stokes, and Brinkman equations were applied. Furthermore, with a Reynolds number (Re) of approximately 1, the flow was assumed to be laminar, allowing inertial terms to be disregarded. Cellular oxygen consumption was also included, and although PDMS is gas-permeable, all wall boundaries were treated as impermeable since the bioreactor was enclosed by two gas-impermeable poly(methyl methacrylate) (PMMA) layers. The results revealed a laminar flow regime with a parabolic velocity profile at a perfusion rate of 5 µL/min, ensuring sufficient oxygen delivery to the HCC cells and preventing hypoxic conditions within the device.

The replication of the liver structure and function has also been explored by some researchers. Chen et al. (2021) created a microfluidic device to mimic the liver lobule structure and tested different GelMA concentrations (5%, 10%, and 15%) (Fig. 4(b)). Computational simulations were used to demonstrate how a strong electric field traps cells guiding cells into precise configurations between electrode layers to mimic liver lobule structures. The results showed that cells co-cultured in 5% GelMA exhibited the highest urea secretion and maintained a liver-lobule-like pattern, demonstrating that this microenvironment effectively preserves liver function and cell pattern despite variations in growth rates and medium flow. On the other hand, Liu et al. (2022b) developed a biomimetic LoC with vascularized liver tissue, a Tri-Vascular Liver-on-a-Chip (TVLOC), which includes a hepatic artery, a portal vein, and a central vein (Fig. 4(c)). Furthermore, the authors developed a bilayer microsphere-generating microsystem with distinct cell types. These bilayer microspheres were then co-cultured with endothelial cells within the TVLOC to form vascularized liver tissue. The results demonstrated that the TVLOC effectively mimics the liver microenvironment's substance concentration gradients and the bilayer microspheres successfully created a structured 3D liver tissue with endothelial cells. Computational tools were used to optimize the microwell size and flow rate and understand oxygen and glucose gradients over time. To this end, they simulated the hepatic artery and portal vein using two inlets, and one outlet representing the central vein. To analyze the nutrient concentration gradients resulting from the convergence of these flows, they developed a model in COMSOL software, and made several assumptions, namely that cell metabolites have a negligible impact on flow velocity, flow behavior is Newtonian, the properties of the medium match those of water at 37 °C, and the impact of cells on ECM porosity is negligible. At the inlet of the hepatic artery, the oxygen partial pressure was set to 90 mmHg and the glucose concentration to 1.0 g/L. At the inlet of the portal vein, the oxygen partial pressure was set to 30 mmHg with a glucose concentration of 4.5 g/L. The diffusion coefficients used were 2.92 × 10^–9^ m^2^/s for oxygen and 9 × 10^–10^ m^2^/s for glucose. For the boundary conditions, a uniform velocity at the inlets was considered (corresponding to 200 µL/h), while at the outlet, the pressure was set to 0 Pa, with a zero-flux concentration condition. Their model successfully maintained hepatocyte function and mimicked the liver microenvironment, demonstrating the utility of combining experimental and computational approaches. Özkan et al. (2023) introduced a 3D vascularized HCC-on-a-chip model to study the therapeutic efficacy of Dox under cirrhotic and inflamed cancer stages (Fig. 4(d)). The model effectively replicated the liver sinusoid's structure by employing concentric layers of cells and a collagen matrix. Findings indicated that increasing inflammation and vascular permeability, due to elevated inflammatory cytokines, led to variability in treatment outcomes. Specifically, enhancing CYP3A4 activity and using TACE to deliver Dox improved therapeutic responses and reduced the increase in endothelial porosity caused by chemotherapy. The model effectively showed how TME factors influence vascular properties, treatment efficacy, and drug delivery strategies. In this study, computational simulations were used to investigate shear stress (SS). They conducted flow simulations using COMSOL Multiphysics software, applying Stokes' Law to quantify flow within the vasculature. They assumed that the fluid had constant viscosity, was incompressible, and that Re was low, allowing them to neglect inertial terms. The simulations were run considering a constant flow rate of 59 μL/min at the inlet and a zero-gauge pressure at the outlet, with a vessel diameter of 435 µm. Additionally, they modeled flow in the ECM using Darcy’s Law, assuming isotropic tissue mechanical properties, considering a collagen porosity of 0.49 and a hydraulic permeability of 10 × 10^–15^ m^2^. The resulting flow profile was then used to calculate the SS at the vessel walls, with the endothelial layer thickness set equal to 15 µm and the thickness of the space equal to 138 µm. Oxygen consumption by the cells was also considered in their simulations. The authors verified that the aligned vascular endothelium was achieved by flowing culture medium at a SS of 1 dyn/cm^2^ over 3 days, replicating the conditions of the human liver tumor microenvironment.

Wu and co-workers (2022) developed a “digital OoC” system that integrates a microwell array with cellular microspheres to enhance parallel processing capabilities compared to traditional single-chamber OoC models, and 2D culture in 24-well plates. The microspheres prepared through electrospray ensure precise control over size and shape, addressing issues related to variability and contamination in drug testing. This new system can accommodate up to 127 uniform liver cancer microspheres as separate analytical units, allowing for consistent and rapid analysis. Note that both digital and traditional OoC models tested were maintained with medium perfusion at a flow rate of 0.25 mL/min. The digital platform demonstrated effective anti-cancer activity for sorafenib and showed better alignment with *in vivo* results compared to the other tested models. Herein, computational simulations were used to study the diffusion of drugs like sorafenib and interferon-γ (IFN-γ) in 2% sodium alginate and compared the diffusion rates in two types of OoC systems, a traditional “single pot” model and a "digital OoC". For the numerical simulation, they used the diffusion coefficients of sorafenib (6.4 × 10^–10^ m^2^/s) and IFN-γ (1.0 × 10^–10^ m^2^/s) in 2% sodium alginate. The initial concentrations of sorafenib and IFN-γ were set at 10 μmol/L and 50 μg/mL, respectively. They employed the convection–diffusion equation to quantitatively analyze the diffusion rates of both small molecules (sorafenib, MW < 10^4^) and large molecules (IFN-γ) in both types of devices. MATLAB software was utilized to solve the convection–diffusion equation. The results revealed that sorafenib, reached a steady-state concentration in the "digital OoC" within 17.3 s, whereas it took 215.9 s to achieve the same concentration in the traditional “single pot” OoC. For larger molecules like IFN-γ, diffusion reached equilibrium in 94.7 s in the "digital OoC", compared to 1594.3 s in the traditional model. This indicates that the "digital OoC" allows for significantly faster diffusion and equilibrium of both small and large molecules compared to the traditional setup. Bhise et al. (2016) developed a LoC supporting the long-term culture of 3D printed hepatic spheroids in GelMA and *in situ* monitoring. Over 30 days, the constructs maintained their functionality, as shown by liver-specific protein secretion and hepatocyte marker staining. The platform effectively mimicked acetaminophen toxicity observed in animal and *in vitro* models, demonstrating its potential for accurate drug toxicity assessment. Computational tools were employed to study oxygen concentration and different flow rates within a cell culture chamber, considering the porosity of hydrogel constructs. In this study, a no-slip boundary condition was applied at the walls, and the convection–diffusion equation was solved to model fluid flow and oxygen transport within the channels and chamber. Although PDMS is oxygen-permeable, all boundaries were treated as impermeable surfaces due to the PMMA layer covering the bioreactor. The hydrogels were modeled as porous media with a uniform volumetric oxygen consumption rate characterized by Michaelis–Menten kinetics, based on the total number of encapsulated cells. A constant and uniform oxygen concentration was specified at the inlet. The diffusion coefficient of oxygen in aqueous media at 37 °C was assumed to be approximately 3.8 × 10^–9^ m^2^/s. Furthermore, an unstructured 3D mesh was used and tested for grid independence, with results showing that a grid size of 800,000 nodes was sufficient to ensure independence from mesh size. Simulations showed that at a 200 µL/h flow rate, the minimum oxygen concentration dropped from 91% of the inlet value on day 1 to an average of 38% (with a minimum of 19%) on day 30. Flow rates higher than 200 µL/h maintained adequate oxygen levels for hepatocytes, thus a flow rate of 200 μL/h was used in subsequent experiments. A different study was presented by Docci et al. (2022). The researchers developed and explored a LoC system, PhysioMimix, to improve the *in vitro* study of drug metabolism and reduce reliance on animal testing. They assessed the system's ability to simulate liver functions by testing how well it mimics the metabolism of various drugs and how effectively it predicts drug behavior in the human body. Mathematical modeling was used to evaluate two different models, a linear model and an evaporation model, to estimate the intrinsic clearance (CLint), the rate at which the liver can metabolize a drug when it has unrestricted access to it, from observed *in vitro* data, considering experimental factors like evaporation, cell number, and sampling volume. Besides that, they utilized both models for a preliminary *in silico* simulation assessment to evaluate the adequacy of the two analyses. The evaporation model was specifically applied to generate *in vitro* concentration-time profiles across a range of theoretical *in vitro* CLint values, along with different evaporation rates. Regarding the numerical simulations, a constant number of cells (N) was considered and incorporated random effects for N and evaporation rate (coefficient of variation equal to 20% for both cases), reflecting their experience with experimental variability. A sampling volume of 18 μL was considered, and to assess variability, they simulated 1,000 concentration-time profiles using the evaporation model based on the previously described conditions. From this full dataset, they randomly sampled three replicates at various time points up to 96 h, repeating this random sampling 100 times. They estimated CLint values by fitting the data with either the linear model or the evaporation model. They then calculated the accuracy of the estimated mean CLint values using the root-mean-square error and assessed bias with the absolute average fold error. Additionally, they evaluated the median uncertainty from 100 experiments and determined the percentage relative standard error. A percentage greater than 30% was considered the threshold for CLint uncertainty. The integration of mathematical modeling with experimental data enhanced the estimation of drug metabolism and provided insights into the performance of the LoC system, demonstrating its potential for generating high-quality and relevant data for drug development.

Table 2 provides an overview of the studies previously discussed, highlighting the culture models developed, cell types used, study aims, the purpose of computational simulations, the software utilized, and whether the authors validated their computational models.

**Table 2:** Summary of reviewed papers detailing cell culture models, cell types, study aims, devices used, computational analysis, software employed, and if validation of the computational models was conducted or not

Cell culture model	Cell types	Study aims	Computational analysis	Software	Validation of the computational model	Ref
Spheroids and coculture of cells within a fibrin gel	RFP-HUVECs^1^, Lung fibroblasts HepG2^2^ cells, Hepatocellular carcinoma cells, HUVECs^3^	Study hypoxic stress	Oxygen transport	Not mentioned	n.a*	Lim et al. 2021
Cells cultured in the microfluidic device coated with fibronectin	Primary rat hepatocytes	Study the role of oxygen and hypoxia‐associated molecules	Oxygen and sodium sulfite transport	COMSOL®	Yes	Kang et al. 2020; Kang et al. 2018
Cells seeded in a device pre-coated with fibronectin and collagen	Huh7^4^	Monitor albumin	Oxygen distribution	COMSOL®	n.a	Kim et al. 2023
Cell suspension injected into the microfluidic device	hESCs^5^ (MShef-3 and HES2)	Determine if an oxygen gradient, generated *in vitro* using their device, can guide functional metabolic zonation during hESC differentiation	Check the spatial control of the oxygen concentration	COMSOL®	Yes	Tonon et al. 2019
Matrigel-coated channels with cancer and heart cells introduced into separate chambers	HepG2 Cells and hCMs^6^	Simulate the side effects of anti-cancer drugs in the heart	Pressure threshold of PDMS membrane	CoventorWare®	Yes	Kamei et al. 2017
Cells cultured on a collagen-coated glass surface and within a hydrogel matrix (GelMA)	C3A Cells and 3T3 Cells	Mimic the structure and function of a liver lobule	Electric field/concentration gradient simulation	COMSOL®	n.a	Chen et al. 2021
Bilayer microspheres with HCs^7^ in the inner layer, LSECs^8^ in the outer layer, and HSCs^9^ encapsulated in collagen surrounding the microspheres	HCs, HSCs, LSECs	Reconstruct the tissue-tissue interfaces based on bilayer microspheres and form vascularized liver tissue	Fluid and oxygen distribution and glucose concentration	COMSOL®	n.a	Liu et al. 2022b
Two-layer concentric structure	HepG2-CYP3A4 Cells, LX-2 Cells, HSCs, THP-1^10^, Microvascular ECs^11^	Understanding the effects of inflammation and cirrhosis on the regulation of drug metabolism during the progression of HCC	Shear stress	COMSOL®	n.a	Özkan et al. 2023
Cells encapsulated in GelMA	Human HepG2 and HCC^12^ cells	Investigate HCC-bone metastasis	Oxygen transport and fluid flow analysis	COMSOL®	n.a	Sharifi et al. 2020
Cells within uniform microspheres maintained in a 2% sodium alginate solution	HepG2, HUVEC and HFF-1^13^	Assess toxicity in a parallel way	Chemical diffusion of sorafenib and interferon-γ	MATLAB®	n.a	Wu et al. 2022
Spheroids mixed with GelMA bioprinted as liquid droplets	HepG2/C3A cells	Assess drug toxicity	Oxygen transport	COMSOL®	n.a	Bhise et al. 2016
Cells seeded onto the scaffolds of the LC-12 plates	Primary HCs	Assess drug metabolism capability	Estimate intrinsic clearance in LoC experiments	R using RxODE for simulations and nlmixr for model fitting	n.a	Docci et al. 2022

* n.a – Not applicable

^1^ RFP-HUVECs—Red fluorescent protein-labeled human umbilical endothelial cells; ^2^ HepG2—Cell line derived from a human liver carcinoma; ^3^ HUVEGs – Human umbilical endothelial cells; ^4^ Huh7—Cell line from a hepatocellular carcinoma; ^5^ hESCs – Human embryonic stem cells; ^6^ hCMs – Human cardiomyocytes; ^7^ HCs – Hepatocytes; ^8^ HSCs – Hepatic stellate cells; ^9^ LSECs—Liver sinusoidal endothelial cells; ^10^ THP-1 – Human monocyte cells; ^11^ ECs – Endothelial cells; ^12^ HCC – Hepatocellular carcinoma; ^13^ HFF-1 – Human foreskin fibroblasts

## Conclusions and future perspectives

The integration of numerical simulations with experimental approaches has significantly advanced the development of LoC models, particularly in the context of cancer research. These sophisticated models effectively replicate the complex microenvironment of the liver, facilitating precise drug testing and deepening our understanding of liver physiology and disease mechanisms.

The 13 studies analyzed in this systematic review highlight the effectiveness of LoC models in replicating liver structure, function, and the tumor microenvironment. By recreating key factors such as hypoxic conditions, vascular interactions, and metabolic zonation, these models provide valuable insights into tumor progression and treatment responses. They also offer a platform for monitoring liver function under varying conditions. Curiously, although reviewed papers use dynamic conditions and microfluidic technology, most studies still seed cells in two dimensions, highlighting the potential for further innovation in future research. The successful integration of computational simulations has further enabled researchers to enhance device design, predict drug diffusion and concentration gradients, replicate complex liver structures such as the liver lobule, and improve flow conditions. However, the reviewed research articles also revealed certain limitations. Many authors did not mention conducting a mesh study, a critical step in ensuring the accuracy of numerical simulations. This involves refining the mesh and observing the convergence of results. This process ensures that the obtained solutions are independent of the mesh discretization and are accurate representations of the physical phenomena. Similarly, a sensitivity analysis should be conducted to evaluate the impact of varying boundary conditions and material properties on the simulation outcomes. By systematically investigating these factors, the reliability and robustness of computational models can be enhanced. Besides this, only three studies among those reviewed mentioned the comparison between the numerical and experimental outcomes to validate the computational model, which underscores a critical gap in ensuring the reliability and applicability of simulation results. Validation is crucial for confirming that the computational outputs are viable and can be extrapolated to experimental practice. For validating the computational model, experimental approaches could include both cell-free and cell-inclusive setups, each offering distinct insights. Validation without cells, such as fluid flow or mechanical deformation studies in empty OoC channels, enables the assessment of basic model parameters like flow dynamics and structural integrity under controlled conditions. While cellular experiments can provide more representative data for comparison with simulation results, it is essential to recognize that computational models can be firstly assessed for accuracy and potential in the absence of such data. Furthermore, one of the fully reviewed papers did not include any representation of computational results, merely presenting their conclusions without providing the supporting data or analysis. Additionally, while many studies claim device optimization, this is often an improvement rather than the result of the systematic application of optimization methods. Moreover, the computational studies presented typically focus on one or two outputs, with methodologies that are often too generalized and lacking in detail, limiting the ability to replicate these results. This highlights the need for more extensive validation and a deeper exploration of computational tools in future research on LoC devices. Despite these challenges, the combination of advanced experimental and computational techniques has led to the development of more physiologically relevant LoC models.

Future research should prioritize increasing the complexity of LoC models by incorporating additional cell types and organ systems. This would enable more comprehensive studies of multi-organ interactions and systemic drug effects, offering a holistic view of drug metabolism and toxicity. The development of patient-specific LoC models using cells derived from individual patients also holds great promise for personalized medicine, allowing therapies to be tailored to the specific genetic and environmental factors of a patient’s tumor. Moreover, refining computational models to better predict complex biological phenomena, such as cellular responses to dynamic microenvironments and drug interactions, will be crucial. This will likely involve the development of more sophisticated algorithms and the integration of machine learning techniques to handle the large datasets generated by these models. Advancements in scalability and automation will also be necessary to enable high-throughput screening, making these models more accessible and cost-effective for pharmaceutical research. Finally, the integration of advanced sensors and imaging techniques within LoC models will enable real-time monitoring of cellular responses and metabolic changes, providing more detailed insights into the dynamics of the liver microenvironment and improving the predictive power of these models.

Addressing these challenges will allow the field of LoC research to continue evolving, ultimately leading to more accurate and reliable models for studying liver cancer and other diseases, and thereby improving therapeutic outcomes.

It should be also noted that a limitation of this systematic review is the potential exclusion of studies where computational models and OoC experiments are not explicitly linked within a single publication. This is particularly relevant for well-established research groups or companies that employ the same OoC design across multiple applications. While internal integration of computational models and OoC experiments might exist, it may not be explicitly documented in a single paper, thus hindering their inclusion in this review.

## Data Availability

No datasets were generated or analysed during the current study.
